# Acute pancreatitis caused by impaction of hydatid membranes in the papilla of Vater: a case report

**DOI:** 10.4076/1757-1626-2-7374

**Published:** 2009-07-07

**Authors:** Panagiotis Katsinelos, Grigoris Chatzimavroudis, Kostas Fasoulas, Eustathios Kamperis, Taxiarchis Katsinelos, Sotiris Terzoudis, George Kokonis, Ioannis Patsis

**Affiliations:** Department of Endoscopy and Motility Unit, “G. Gennimatas” General HospitalThessalonikiGreece

## Abstract

Acute pancreatitis is a rare complication of hydatidosis and the successful use of endoscopic sphincterotomy associated with extraction of hydatid membranes has been rarely reported. We describe a young man who developed acute pancreatitis after rupture of an echinococcus cyst, located at the left hepatic lobe, into the biliary tract. The cause of pancreatitis was confirmed by endoscopic retrograde cholangiopancreatography, which revealed the presence of a daughter cyst impacted in the major papilla. After sphincterotomy and removal of hydatid membranes from the biliary tract, the patient presented rapid resolution of pancreatitis and made an uneventful recovery.

## Introduction

Hydatidosis is a zoonosis that is generally caused by infection with Echinococcus granulosus. The disease is endemic in many countries, including those around the Mediterranean Sea, Central Asia, Far East and Latin America [1]. Liver is the most common site of hydatid cysts and less frequently they can be found in the lungs and much more rarely in the spleen, kidneys, brain, muscles, bone and pancreas [1,2]. Rupture of a hydatid cyst into the biliary tract is rare and is manifested as obstructive jaundice or cholangitis [1,2]. A few cases of acute pancreatitis associated with the presence of hydatid membranes in the biliary tract have been described [3-13].

We herein describe the first case of hydatid acute pancreatitis by documentation of impaction of hydatid membranes in the papillary orifice.

## Case presentation

A 31-year-old Greek farmer was admitted to the Department of Internal Medicine with pain in the epigastric area that had started 3 days earlier. The pain was intense and constant, radiating to the back and associated with nausea and vomiting. There was no history of drug or alcohol consumption.

Clinical examination revealed an acutely ill patient, mildly jaundiced and febrile (38°C) and was remarkable for epigastric tenderness and a palpably enlarged left liver lobe 2 cm below the right costal margin. Laboratory data demonstrated a high leukocyte count of 14.5 × 10^9^/L (normal 4.0-10.0 × 10^9^/L), eosinophils 0.6 × 10^9^/L (normal 0.0-0.5 × 10^9^/L), total bilirubin 47 μmol/L (normal 2-17 μmol/L), alkaline phosphatase 238 IU/L (normal 40-120 IU/L), gamma glutamyl transferase 312 IU/L (normal 20-40 IU/L), asparate aminotransferase 1 IU/L (normal 5-40 IU/L), alanine aminotransferase 178 IU/L (normal 5-40 IU/L), serum amylase 3254 IU/L (normal 30-120 IU/L), lipase 4234 IU/L (normal 20-280 IU/L), and C-reactive protein 38 mg/L (normal <0.5 mg/L).

**Figure 1.**
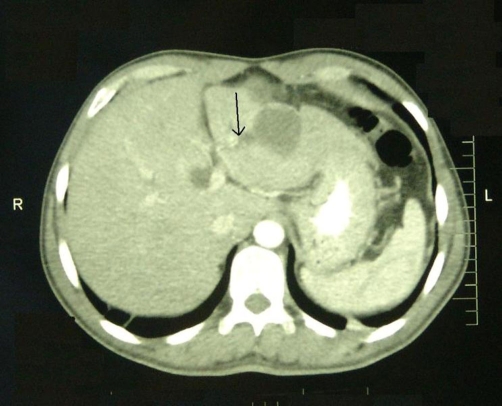
CT of the abdomen revealing a polychorous cyst in the left hepatic lobe with rupture in the biliary tract (arrow).

**Figure 2.**
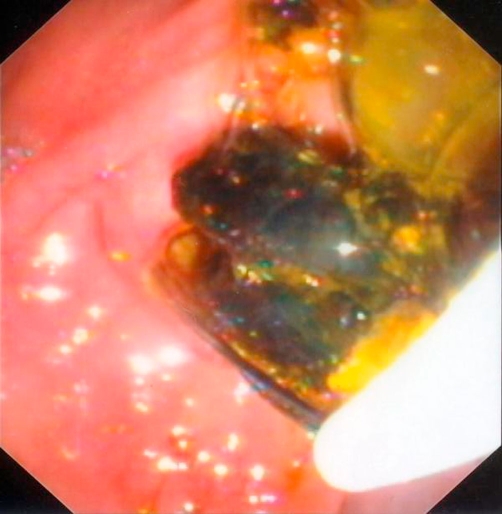
Endoscopic view of hydatid membranes protruding from major papilla before extraction with a Dormia basket.

**Figure 3.**
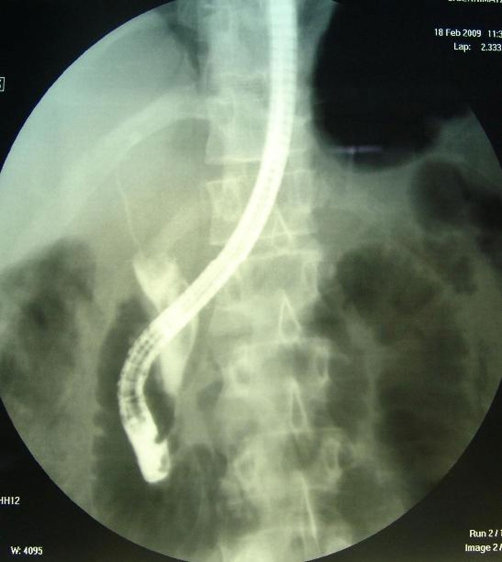
ERCP showing a dilated common bile duct, containing multiple filling defects with irregular morphology.

**Figure 4.**
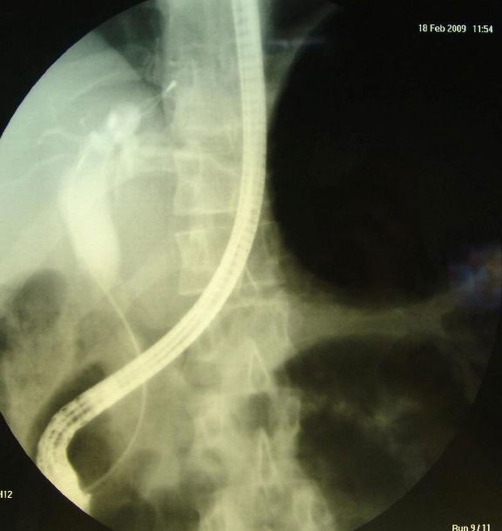
ERCP demonstrating complete removal of hydatid membranes from the biliary tract.

Abdominal computed tomography (CT) showed a large multilocular cyst in the left hepatic lobe with rupture into the biliary tract, dilated main biliary ducts (common bile duct diameter 22 mm) and a diffusely swollen pancreas with indurations around it (Figure 1). Positive ELISA for IgM echinococcal antibodies at 1/1280 dilution and positive echinococcal immunoelectrophoresis confirmed the diagnosis of intrabiliary rupture of the hydatid cyst. An emergent endoscopic retrograde cholangiopancreatography (ERCP) demonstrated the presence of hydatid membranes impacted in the ampulla of Vater and protruded through the papillary orifice (Figure 2) and a dilated common bile duct with multiple filling defects (Figure 3). Endoscopic sphincterotomy was performed and all hydatid membranes were removed via a Dormia basket and balloon (Figure 4). The patient had a rapid resolution of pancreatitis with an uneventful recovery and was referred for elective surgery for the liver hydatid cyst.

## Discussion

A hydatid cyst is normally well tolerated in humans until its development results in pressure on adjacent structures [1]. The cyst may also burst into the peritoneal or thoracic cavity, which may cause anaphylactic shock or give rise to many new cysts [1,2]. Spontaneous rupture of a hepatic hydatid cyst into the biliary tract occurs in 5-17% of cases and can be associated with abdominal pain, jaundice, fever, and cholangitis [1,2]. Serology is useful in confirming the diagnosis of hydatidosis; however a negative serological result does not exclude the diagnosis [14]. About 50% of cases have negative serology, while false-positive results can be caused by cysticercosis [14].

Despite the mechanism of hydatid, pancreatitis is generally considered to be unknown; we believe that it could be explained by the mechanical obstruction of the papillary orifice by daughter cysts resulting in reflux of a mixture of bile and hydatid material into the pancreatic duct and increase of intrapancreatic pressure, which triggers the inflammatory cascade of acute pancreatitis.

Our case is very intriguing because for first time an endoscopic image depicted hydatid membranes impacted in the papillary orifice, thus confirming our hypothesis of acute pancreatitis development. The rapid resolution of pancreatitis after endoscopic sphincterotomy and removal of hydatid membranes further strengthens our hypothesis. The efficacy and safety of endoscopic sphincterotomy for the treatment of hydatid cysts with rupture into the biliary tract is well established [15,16]. However, the successful use of this technique in the treatment of pancreatitis associated with hepatic hydatidosis has been described previously in few cases [5,6,11,12].

We referred our patient for surgical extirpation of hepatic hydatid cyst because surgery remains the treatment of choice [1,2], despite long-term (3 months or more) treatment with benzimidazoles (mebendazole, albendazole) has also shown efficacy against the cystic stage [17-19]. Recent literature suggests that cautious percutaneous drainage with concurrent administration of antihelminthic therapy may be an effective alternative to surgical intervention in selected cases [20].

## Conclusion

Our case demonstrates that the cause of acute pancreatitis, after rupture of a hepatic echinococcus cyst into the biliary tract, is the impaction of hydatid membranes in the papillary orifice. Endoscopic sphincterotomy and removal of membranes is an effective and safe treatment.

## List of abbreviations

CT: Computed Tomography
ERCP: Endoscopic Retrograde Cholangiopancreatography
